# Establishment and Application of an Elastic–Plastic Damage Constitutive Model for Ceramic Fiber Insulation Tiles

**DOI:** 10.3390/ma17246094

**Published:** 2024-12-13

**Authors:** Yiming Wang, Yesheng Zhong, Yining Huang, Xiaoliang Ma, Liping Shi, Xiaodong He

**Affiliations:** 1 Center for Composite Materials and Structures, Harbin Institute of Technology, Harbin 150080, China; 21b918026@stu.hit.edu.cn (Y.W.); zhongyesheng@hit.edu.cn (Y.Z.); hexd@hit.edu.cn (X.H.); 2 College of Materials Science and Chemical Engineering, Harbin Engineering University, Harbin 150001, China; zhoudu99@126.com

**Keywords:** constitutive model, damage factor, ceramic fiber insulation tile, UAMT

## Abstract

A thermal protection system is critical for ensuring the safe take-off and return of various aircraft. A key heat-resistant material within this system is the ceramic fiber insulation tile (CFIT), which is a porous three-dimensional network material with density ranges from 0.3 to 0.4 g/cm^3^ that exhibits complex mechanical behaviors. Due to the complexity of the service environment, experimental methods cannot accurately capture the mechanical behavior of a CFIT. Although simulation-based methods can provide insights, an accurate constitutive model for CFITs has yet to be established. To predict its complex mechanical behavior, an elastic–plastic damage constitutive model was established for CFITs. Based on the Hashin criteria and four fundamental assumptions, a yield rule was modified by introducing a damage factor in the TTT direction. The model was encoded into a user–material subroutine (UAMT) integrated within ABAQUS to capture the mechanical responses under four typical working conditions. The change trend of the simulation curve closely aligned with that of the experiment curve, better characterizing the stress–strain relationship of the CFIT under different working conditions such as compression, tension, and shear and the error was less than 18%. The proposed approach was validated by designing a millimeter-level indentation experiment. The results in this paper demonstrate that the maximum loading depths of the simulation and experiment were consistent, and the relative errors were within 12%, respectively. The research provides a reliable elastic–plastic damage constitutive model to predict the mechanical behavior of CFITs under complex working conditions.

## 1. Introduction

A ceramic fiber insulation tile (CFIT), as one type of transversely isotropic material, is lightweight and has high strength and low thermal conductivity. It is widely used in the thermal protection systems of hypersonic aircraft [1,2,3,4,5]. Hypersonic aircraft experience multiple flight stages during service, including launch, on-orbit operation, return, autonomous flight, and landing [6,7]. During these different service stages, the surface materials of a spacecraft need to endure the coupled effects of force, heat, and vibration as well as the impacts of space debris. However, a CFIT is an intrinsically brittle material under high-temperature conditions, so it is important to simulate the mechanical behavior of CFITs in extreme environments [8,9,10].

The premise of mechanical behavior simulation is to establish a suitable constitutive model [11,12]. To date, many researchers have proposed constitutive models of CFITs at the microscopic scale; these constitutive models are used to design and prepare the CFIT [13,14]. Li et al. [15,16] used experimental, micromechanical, and finite element methods to study the compressive behaviors of three-dimensional random fibrous material in different directions. Long et al. [17,18] constructed a three-dimensional finite element model to study the through-the-thickness compressive and interfacial shear performance of ceramic fiber insulation tiles (CFITs) and some of the factors affecting them. However, the size and number of fibers in these models are limited, so it is difficult to simulate the mechanical behaviors of these tiles under complex loads [19]. Another, more reliable, method is to establish macroscopic constitutive models to identify the mechanical behavior under complex loads [20]. Generally, a macroscopic constitutive model is constructed using the finite element method. However, it is necessary to modify the yield, flow, and damage rules according to the specific properties of the materials [21,22].

The mechanical behavior of a CFIT is similar to wood, both of which are transversely isotropic materials [23,24]. For wood, the Hashin criteria [25,26] are used to produce a macroscopic elastic–plastic constitutive model, but a constitutive study does not consider the mechanical response under tensile and shear loads [27,28,29]. Tagarielli et al. [30] developed an elastic–plastic macroscopic constitutive model with a quadratic yield surface for wood. The proposed model was determined using a combination of compressive and shear tests and was used to predict the indentation response of wood to a conical indenter. The compressive and tensile properties of CFITs are similar to those of wood, but the constitutive model of wood does not consider the influence of shear properties. Thus, considering the damage process, a macroscopic constitutive model was established by introducing the damage factor to modify the yield criterion to calculate the complex mechanical properties of CFITs.

In this study, an elastic–plastic damage constitutive model of a CFIT was established, and this was examined in combination with the results of mechanical experiments. Indentation tests with a millimeter-level indenter were used to obtain load–displacement curves under compression–shear loading. The user–material (UMAT) subroutine was integrated into ABAQUS (v.6.14) to calculate the mechanical response of the materials under compression, tension, shear, and indentation loads. By comparing the results of the experiments and simulations, the effectiveness of the elastic–plastic damage constitutive model could be verified, providing a model and theory to study the mechanical behavior of transversely isotropic materials in complex environments.

## 2. Materials and Parameter Tests

### 2.1. Materials

**Figure 1:**
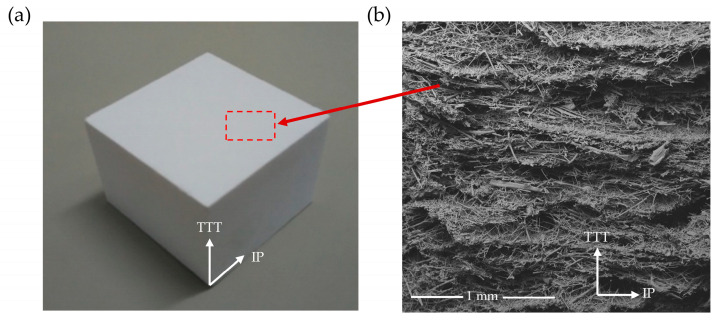
Ceramic fiber insulation tile: (**a**) photograph; (**b**) SEM image showing microstructure. The red area is the top of the sample used for SEM testing.

A CFIT is prepared using an optimized combination of high-purity silica fibers with different aspect ratios and different fillers, dispersing them into a three-dimensional space. After suction filtration and drying, they are sintered under a vacuum using different temperature gradient conditions. The specific raw materials (Shandong Dongheng Guoxian New Materials Co., Ltd., Dongying, China) and temperature gradients are shown in Table 1. This process has the advantage of producing porous materials with a highly connected microstructure. The density ranges from 0.3 to 0.4 g/cm^3^, respectively. These materials are usually used in the sub-high-temperature zones of aircraft, and they show good volume stability at high temperatures. Figure 1 shows a photograph and a scanning electron microscope (SEM) image of a CFIT. The preparation process can lead to different microstructures being formed in the through-the-thickness (TTT) and in-plane (IP) directions. The ceramic fibers are randomly distributed in the IP direction and the ceramic fibers show a layered distribution in the TTT direction. These distributions influence the mechanical properties of the CFIT in the two directions.

### 2.2. Parameter Tests

**Figure 2:**
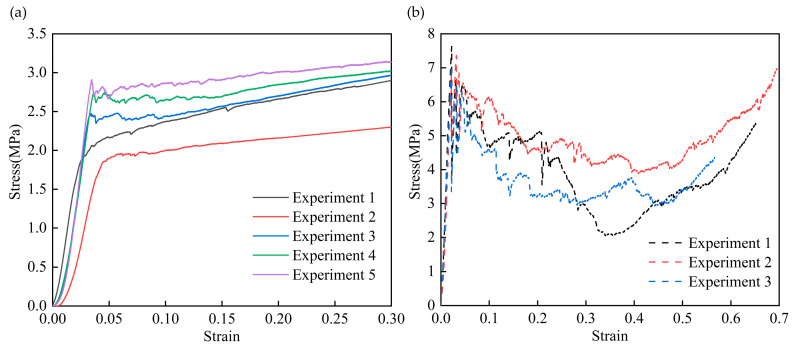
The results of compressive properties: (**a**) in the TTT direction; (**b**) in the IP direction.

We used an Instron 5569 electronic universal testing machine (Instron, Norwood, MA, USA) to investigate the mechanical behavior of the CFIT in terms of compression along the (TTT) and (IP) directions. For the compressive tests, the size of the CFIT samples was 20 × 20 × 20 mm^3^ and the loading rate was 1 mm/min in accordance with the GB/T 1964-2023 standard [31]. The results of these tests are shown in Figure 2. 

It can be seen from Figure 2a that there were two stages of compression in the TTT direction. These were the elastic stage, in which the material produced elastic deformation under compression, and the plastic yield stage, in which the stress exhibited the characteristics of progressive damage. For the TTT direction, the internal fiber framework underwent elastic deformation at the initial stage, which is a part of the linear elastic stage and determines the impact resistance of a material. As the load was greater than the fiber strength in the fiber skeleton, the curve entered the yield platform stage. At this time, cracks began to form in the fiber skeleton and then broke after expansion. The fracture process of the crack continuously absorbed external energy to ensure that the stress–strain curve remained in the platform stage. As the load continued to increase, the material skeleton gradually failed, causing the porous structure to be destroyed. The pores between the fibers were filled with broken fibers and the material gradually became densified. The average compressive strength from five sets of experiments was found to be 2.50 ± 0.51 MPa and the average compressive modulus was 115.54 ± 16.74 MPa in the TTT direction.

Figure 2b shows the results of the compressive properties in the IP direction. The average compressive strength from three sets of experiments was found to be 7.4 ± 0.32 MPa, with an average compressive modulus of 380 ± 2.48 MPa. Thus, there was concordance between the microstructure and compressive properties in the TTT and IP directions, and we determined that the CFIT was a transversely isotropic material.

**Figure 3:**
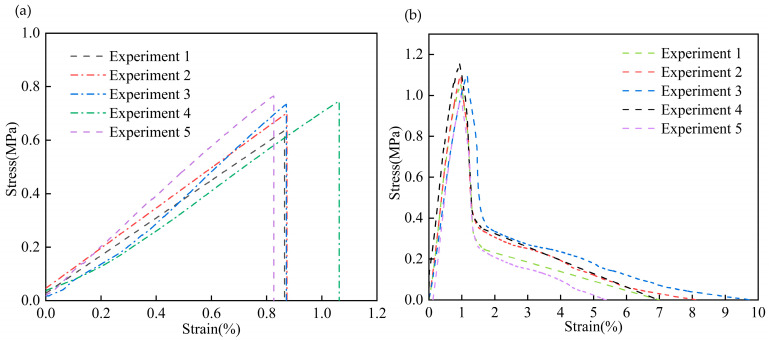
Results of mechanical tests in the TTT direction: (**a**) tensile properties; (**b**) shear properties.

To accurately obtain the constitutive parameters of the CFIT, its tensile and shear properties were characterized using an Instron 5569 machine in the TTT direction. For the tensile tests, the size of the CFIT was 50 × 50 × 60 mm^3^ and the loading rate was 0.5 mm/min, in accordance with the GB/T 1452-2018 standard [32]. For the shear tests, the size of the CFIT samples was 80 × 40 × 10 mm^3^ and the loading rate was 0.5 mm/min, in accordance with the GB/T 10007-2008 standard [33]. Five sets of parallel experiments were conducted for both tensile and shear tests. Figure 3 presents the results of the mechanical tests in the TTT direction.

It can be seen from Figure 3a that the material showed a trend of brittle fracture after reaching its elastic limit under the action of a tensile load. For the TTT direction, the average tensile strength was found to be 0.78 ± 0.25 MPa, with an average tensile modulus of 62.46 ± 15.28 MPa. The shear results are shown in Figure 3b. In the shear stress–strain curves, it can be seen that when the shear stress reached a maximum value, the material experienced a brittle fracture. The subsequent slow decline was caused by the destruction of the material’s internal structure under the load. For the TTT direction, the average shear strength was found to be 1.12 ± 0.09 MPa, with an average shear modulus of 126.18 ± 15.39 MPa. The specific properties of the CFIT are summarized in Table 2.

## 3. Elastic–Plastic Damage Constitutive Model

Considering the research of Zhang et al. [34,35,36] and Tagarielli et al. [30] on transversely isotropic constitutive models, and combined with the unique microstructure of the CFIT, an elastic–plastic damage constitutive model was established based on the framework of classical elastoplastic mechanics. This included the following five parts: a basic assumption, the elastic property, a yield rule, a damage reinforcement rule, and a plastic flow rule.

### 3.1. Fundamental Assumptions

The strength and stiffness values of a CFIT are different in different directions, and there are also differences in the tensile and compressive strengths in the same direction. The responses of this material to different loads are also different. For example, its performance under pressure is mainly ductile, and its failure mode under tension and shear is brittle. As such, the mechanical properties have obvious complexity. According to the framework of classical elastoplastic mechanics, the elastic–plastic damage constitutive model was based on the following fundamental assumptions:

(1) In the elastic stage, the CFIT is an ideal transversely isotropic material;
(2) The yield rule of the material complies with simplified Hashin criteria;
(3) The CFIT behaves as an ideal linear elastic material before tensile and shear failure, and brittle fracture occurs after tensile and shear failure;
(4) During compression, the CFIT behaves as an ideal linear elastic material before yielding; it enters a hardening stage after yielding and it experiences an entirely plastic flow after reaching the final yield surface.

### 3.2. Elastic Property

According to the assumption of transverse isotropy, a generalization of Hooke’s law is used to describe the stress–strain relationship of the CFIT in the elastic range, as follows:

(1) $$\left[\begin{array}{c} \Delta \sigma_{11}\\ \Delta \sigma_{22}\\ \Delta \sigma_{3}\\ \Delta \tau_{12}\\ \Delta \tau_{23}\\ \Delta \tau_{13} \end{array}\right]=\mathrm{DDSDDE}\left[\begin{array}{c} \Delta \varepsilon_{11}\\ \Delta \varepsilon_{22}\\ \Delta \varepsilon_{33}\\ \Delta \gamma_{12}\\ \Delta \gamma_{23}\\ \Delta \gamma_{13} \end{array}\right]=\left[\begin{array}{cccccc} \frac{E_{11}\left(1-v_{23}v_{32}\right)}{\delta } & \frac{E_{11}\left(v_{21}+v_{31}v_{23}\right)}{\delta } & \frac{E_{11}\left(v_{31}+v_{21}v_{23}\right)}{\delta } & 0 & 0 & 0\\ \frac{E_{22}\left(v_{12}+v_{32}v_{13}\right)}{\delta } & \frac{E_{22}\left(1-v_{13}v_{31}\right)}{\delta } & \frac{E_{22}\left(v_{32}+v_{12}v_{31}\right)}{\delta } & 0 & 0 & 0\\ \frac{E_{22}\left(v_{13}+v_{23}v_{12}\right)}{\delta } & \frac{E_{22}\left(v_{23}+v_{13}v_{21}\right)}{\delta } & \frac{E_{22}\left(1-v_{12}v_{21}\right)}{\delta } & 0 & 0 & 0\\ 0 & 0 & 0 & G_{12} & 0 & 0\\ 0 & 0 & 0 & 0 & G_{23} & 0\\ 0 & 0 & 0 & 0 & 0 & G_{13} \end{array}\right]\left[\begin{array}{c} \Delta \varepsilon_{11}\\ \Delta \varepsilon_{22}\\ \Delta \varepsilon_{33}\\ \Delta \gamma_{12}\\ \Delta \gamma_{23}\\ \Delta \gamma_{13} \end{array}\right]$$

where DDSDDE is the Jacobian matrix; $\delta =1-\nu_{12}\nu_{21}-\nu_{23}\nu_{32}-\nu_{13}\nu_{31}-{2\nu }_{21}\nu_{32}\nu_{13}$; $E_{11}$ is the elastic modulus perpendicular to the isotropic plane (the TTT direction); $E_{22}$ is the elastic modulus parallel to the isotropic plane; $\nu_{12}{,\;\nu }_{23}{,\;\mathrm{and}\;\nu }_{13}$ represent the three positive Poisson ratios; $\nu_{21}{,\;\nu }_{32}{,\;\mathrm{and}\;\nu }_{31}$ represent the three negative Poisson ratios; Δε is the strain increment; Δσ is the normal stress increment; Δγ is the shear strain increment; and Δτ is the shear stress increment.

As a CFIT is a type of transversely isotropic material, then

(2) $$\nu_{12}\;{=\nu }_{13}\;G_{12}{=G}_{13}$$

Theoretically, a transversely isotropic material should contain

(3) $$\nu_{23}=\frac{E_{22}}{{2G}_{23}}-1$$

However, the ceramic *ν*_23_, calculated from the experimental value, was a negative number when using Equation (3) and could not reflect the actual situation, so Equation (3) was abandoned. Based on the above theory, the CFIT possessed the following elastic performance parameters: *E*_11_, *E*_22_, *ν*_12_, *ν*_23_, *G*_12_, and *G*_23_.

### 3.3. Yield Rule

For transversely isotropic materials such as CFITs, the simplified Hashin criteria are used to establish a constitutive model. According to these rules, the failure modes of fiber material can be divided into two types. Their yield function for the IP direction is as follows:

(4) $$f_{IP}=\frac{I_{1}^{2}}{X^{2}}+\frac{I_{4}^{2}}{S_{L}^{2}}-1;\;X=\{\begin{array}{c} X_{T}\;\;\;\;\mathrm{if}\;\;\;\;I_{1}>0\\ X_{C}\;\;\;\;\mathrm{if}\;\;\;\;I_{1}<0 \end{array}$$

For the TTT direction, it is

(5) $$f_{TTT}=\frac{I_{2}^{2}}{Y^{2}}+\frac{I_{3}^{2}}{S_{T}^{2}}-1;\;Y=\{\begin{array}{c} Y_{T}\;\;\;\;\;{\mathrm{if}\;I}_{2}>0\\ Y_{C}\;\;\;\;{\mathrm{if}\;I}_{2}<0 \end{array}$$

(6) $$I_{1}{=\sigma }_{11};\;I_{2}{=\sigma }_{22}{+\sigma }_{33};\;I_{3}={\sigma_{23}}^{2}{-\sigma }_{22}\sigma_{33}\;I_{4}={\sigma_{12}}^{2}+{\sigma_{13}}^{2};\;I_{5}{=2\sigma }_{12}\sigma_{13}\sigma_{23}{-\sigma }_{22}{\sigma_{31}}^{2}{-\sigma }_{33}{\sigma_{12}}^{2}$$

This yield criterion, assuming four different failure modes (IP-direction tensile failure, IP-direction compressive failure, TTT-direction tensile failure, and TTT-direction compressive failure), can be used to reflect the properties of materials with different tensile and compressive strengths.

### 3.4. Damage Reinforcement Rule

The complete plastic flow of a CFIT is achieved by gradual strengthening during compression. In this work, the coefficients of $N_{L}$ and $N_{T}$ were used to reflect the transfer of the yield surface in the IP and TTT directions, respectively. For compression in the IP direction, the initial yield equation of the material can be expressed as follows:

(7) $$f_{IP}=\frac{I_{1}^{2}}{\left({1-N}_{L}\right)X^{2}}+\frac{I_{4}^{2}}{S_{L}^{2}}-1\;\;\;\;X=\{\begin{array}{c} X_{T}\;\;\;\;\mathrm{if}\;\;\;\;I_{1}>0\\ X_{C}\;\;\;\;\mathrm{if}\;\;\;\;I_{1}<0 \end{array}$$

In the TTT direction, it is

(8) $$f_{TTT}=\frac{I_{2}^{2}}{\left({1\;-\;N}_{T}\right)Y^{2}}+\frac{I_{3}^{2}}{S_{T}^{2}}-1\;\;\;\;Y=\{\begin{array}{c} Y_{T}\;\;\;\;\;{\mathrm{if}\;I}_{2}>0\\ Y_{C}\;\;\;\;{\mathrm{if}\;I}_{2}<0 \end{array}$$

The damage failure coefficient in the TTT direction is

(9) $$F=\frac{\varepsilon_{1}}{\varepsilon_{0}}\;\;{\;\mathrm{if}\;\varepsilon }_{1}>0$$

Compared with the constitutive model of wood material, the constitutive model of a CFIT considers progressive damage in the TTT direction. The damage variable in the TTT direction is as follows:

(10) $$D_{v}=1-exp\left[100\;\text{×}\;\left(1-F\right)\right]\;D_{i+1}{=D}_{v}{\;\;\;\mathrm{if}\;\;\;D}_{v}{>D}_{i}$$

In the above equations, $\varepsilon_{0}$ is the strain of the damage failure, which is related to the strength and modulus. $D_{v}$ is the damage variable at step *V*. $D_{i}$ is the damage variable at step i. $D_{i+1}$ is the damage variable at step *i* + 1. The maximum value of *D* is 1 and the minimum value of *D* is zero.

According to Equation (10) and considering Equations (6) and (7), the equations of failure for the tensile and shear properties were obtained. For the IP direction, these were as follows:

(11) $$f_{IP}=\frac{I_{1}^{2}}{\left({1-N}_{L}\right)X^{2}}+\frac{I_{4}^{2}}{S_{L}^{2}}-1\;\;\;\;X=\{\begin{array}{c} X_{T}\;\;\;\;\mathrm{if}\;\;\;\;I_{1}>0\\ X_{C}\;\;\;\;\mathrm{if}\;\;\;\;I_{1}<0 \end{array}$$

For the TTT direction, they were as follows:

(12) $$f_{TTT}=\frac{I_{2}^{2}}{\left({1-N}_{T}\right)\left({1-D}_{n}\right)Y^{2}}+\frac{I_{3}^{2}}{S_{T}^{2}}-1\;\;\;\;Y=\{\begin{array}{c} Y_{T}\;\;\;\;\;{\mathrm{if}\;I}_{2}>0\\ Y_{C}\;\;\;\;{\mathrm{if}\;I}_{2}<0 \end{array}$$

where the calculation formulas for $I_{1}$, $I_{2}$, $I_{3}$, $I_{4}$, and $I_{5}$ were the same as in Equation (5).

### 3.5. Plastic Flow Rule

**Figure 4:**
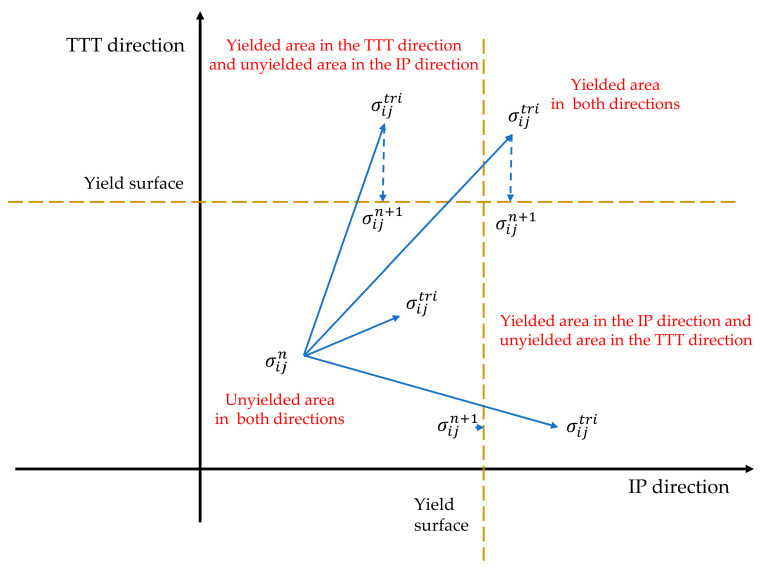
Stress update process of plastic flow.

After the mechanical behavior of a CFIT enters the plastic flow stage, the stress of the materials remains unchanged and the strain continues to increase. The plastic flow process of the model is suitable for the radial return algorithm. When exceeding it, the stress returns to the yield surface. Figure 4 illustrates the stress update process of plastic flow, and its specific description is as follows. First, a trial stress is calculated using the strain increment by assuming that the stress tensor after the nth iterative calculation is $\sigma_{ij}^{\mathrm{tri}}$, as follows:

(13) $$\sigma_{\mathrm{ij}}^{\mathrm{tri}}{=\sigma }_{\mathrm{ij}}^{n}{+D}_{\mathrm{ijkl}}{d\varepsilon }_{\mathrm{kl}}$$

where $D_{\mathrm{ijkl}}$ is the updated Jacobian matrix.

The trial stress is substituted into the yield equation. If ${f(\sigma }_{\mathrm{ij}}^{\mathrm{tri}})\;\leq \;0$, $\sigma_{\mathrm{ij}}^{\mathrm{tri}}$ does not exceed the yield surface; this means that the material is still in an elastic state. The stress at step *n* + 1 is then

(14) $$\sigma_{\mathrm{ij}}^{n+1}{=\sigma }_{\mathrm{ij}}^{n}$$

If ${f(\sigma }_{\mathrm{ij}}^{\mathrm{tri}})\;\geq \;0$, $\sigma_{\mathrm{ij}}^{\mathrm{tri}}$ exceeds the yield surface; this means that the material has begun to yield. As the trial force is not the real material stress, it is necessary to remove the part beyond the yield surface from the trial stress to return the stress state to the yield surface. In the UMAT subroutine, the stress update for the material is determined by the transfer of the yield surface in two directions. It can be seen from Figure 4 that the stress of step *n*+1 returned to the yield surface by removing the excess stress to realize the stress update.

The total strain increment can be expressed as the sum of the elastic strain increment and plastic strain increments.

(15) $${d\varepsilon }_{\mathrm{ij}}{=d\varepsilon }_{\mathrm{ij}}^{e}{+d\varepsilon }_{\mathrm{ij}}^{p}$$

The real stress of the material is as follows:

(16) $$\sigma_{\mathrm{ij}}^{n+1}{=\sigma }_{\mathrm{ij}}^{\mathrm{tri}}{-D}_{\mathrm{ijkl}}{d\varepsilon }_{\mathrm{kl}}^{p}$$

According to the relevant flow rule, the formula for the plastic strain increment is as follows:

(17) $${d\varepsilon }_{\mathrm{ij}}^{p}=\Delta \lambda \frac{\partial f(\sigma )}{\partial \sigma_{\mathrm{ij}}}$$

Δλ is the consistency coefficient in the plastic flow rule, which is different in the IP and TTT directions.

The plastic strain increment is the partial derivative of the yield function ‘*f*’ with respect to the stress component ‘σ’. The plastic strain increments are as follows:

(18) $${d\varepsilon }_{11}^{p}=\Delta \lambda_{\mathrm{IP}}\frac{\partial f_{\mathrm{IP}}}{\partial \sigma_{11}}\;,\;{d\varepsilon }_{12}^{p}=\Delta \lambda_{\mathrm{IP}}\frac{\partial f_{\mathrm{IP}}}{\partial \sigma_{12}}\;,\;{d\varepsilon }_{13}^{p}=\Delta \lambda_{\mathrm{IP}}\frac{\partial f_{\mathrm{IP}}}{\partial \sigma_{13}}$$

(19) $${d\varepsilon }_{21}^{p}=\Delta \lambda_{\mathrm{IP}}\frac{\partial f_{\mathrm{IP}}}{\partial \sigma_{21}}\;,\;{d\varepsilon }_{22}^{p}=\Delta \lambda_{\mathrm{TTT}}\frac{\partial f_{\mathrm{TTT}}}{\partial \sigma_{22}}\;,\;{d\varepsilon }_{23}^{p}=\Delta \lambda_{\mathrm{TTT}}\frac{\partial f_{\mathrm{TTT}}}{\partial \sigma_{23}}$$

(20) $${d\varepsilon }_{31}^{p}=\Delta \lambda_{\mathrm{IP}}\frac{\partial f_{\mathrm{IP}}}{\partial \sigma_{31}}\;,\;{d\varepsilon }_{32}^{p}=\Delta \lambda_{\mathrm{TTT}}\frac{\partial f_{\mathrm{TTT}}}{\partial \sigma_{32}}\;,\;{d\varepsilon }_{33}^{p}=\Delta \lambda_{\mathrm{TTT}}\frac{\partial f_{\mathrm{TTT}}}{\partial \sigma_{33}}$$

where $\Delta \sigma_{\mathrm{ij}}$ is the real stress increment of the material, which is caused by the pure elastic strain increment. The value of $\Delta \sigma_{\mathrm{ij}}$ is the trial force increment minus the false stress increment caused by the plastic strain increment. It is expressed as follows:

(21) $$\Delta \sigma_{11}=\Delta \sigma_{11}^{\mathrm{tri}}{-D}_{1111}\Delta \lambda_{\mathrm{IP}}{\frac{\partial f_{\mathrm{IP}}}{\partial \sigma_{11}}|}_{n}{-D}_{1122}\Delta \lambda_{\mathrm{TTT}}{\frac{\partial f_{\mathrm{TTT}}}{\partial \sigma_{22}}|}_{n}{-D}_{1133}\Delta \lambda_{\mathrm{TTT}}{\frac{\partial f_{\mathrm{TTT}}}{\partial \sigma_{33}}|}_{n}$$

(22) $$\Delta \sigma_{12}=\Delta \sigma_{21}=\Delta \sigma_{12}^{\mathrm{tri}}{-2D}_{1212}\Delta \lambda_{\mathrm{IP}}{\frac{\partial f_{\mathrm{IP}}}{\partial \sigma_{12}}|}_{n}$$

(23) $$\Delta \sigma_{13}=\Delta \sigma_{31}=\Delta \sigma_{13}^{\mathrm{tri}}{-2D}_{1313}\Delta \lambda_{\mathrm{IP}}{\frac{\partial f_{\mathrm{IP}}}{\partial \sigma_{13}}|}_{n}$$

(24) $$\Delta \sigma_{22}=\Delta \sigma_{22}^{\mathrm{tri}}{-D}_{2211}\Delta \lambda_{\mathrm{IP}}{\frac{\partial f_{\mathrm{IP}}}{\partial \sigma_{11}}|}_{n}{-D}_{2222}\Delta \lambda_{\mathrm{TTT}}{\frac{\partial f_{\mathrm{TTT}}}{\partial \sigma_{22}}|}_{n}{-D}_{2233}\Delta \lambda_{\mathrm{TTT}}{\frac{\partial f_{\mathrm{TTT}}}{\partial \sigma_{33}}|}_{n}$$

(25) $$\Delta \sigma_{33}=\Delta \sigma_{33}^{\mathrm{tri}}{-D}_{3311}\Delta \lambda_{\mathrm{IP}}{\frac{\partial f_{\mathrm{IP}}}{\partial \sigma_{11}}|}_{n}{-D}_{3322}\Delta \lambda_{\mathrm{TTT}}{\frac{\partial f_{\mathrm{TTT}}}{\partial \sigma_{22}}|}_{n}{-D}_{3333}\Delta \lambda_{\mathrm{TTT}}{\frac{\partial f_{\mathrm{TTT}}}{\partial \sigma_{33}}|}_{n}$$

(26) $$\Delta \sigma_{23}=\Delta \sigma_{32}=\Delta \sigma_{23}^{\mathrm{tri}}{-2D}_{2323}\Delta \lambda_{\mathrm{TTT}}{\frac{\partial f_{\mathrm{TTT}}}{\partial \sigma_{23}}|}_{n}$$

Assuming that step *n* of the material has yielded and that the stress state of step *n* and step *n* + 1 are located on the yield surface, then

(27) $${f\;}^{n}=0\;,\;\;{f\;}^{n+1}=0\;,\;\Delta f=0$$

The first-order Taylor expansion of Formula (27) is then carried out. It is expressed as follows:

(28) $$\Delta f_{\mathrm{IP}}\approx {\frac{\partial f_{\mathrm{IP}}}{\partial \sigma_{11}}|}_{n}\Delta \sigma_{11}+2{\frac{\partial f_{\mathrm{IP}}}{\partial \sigma_{12}}|}_{n}\Delta \sigma_{12}+2{\frac{\partial f_{\mathrm{IP}}}{\partial \sigma_{13}}|}_{n}\Delta \sigma_{13}=0\;\Delta f_{\mathrm{TTT}}\approx {\frac{\partial f_{\mathrm{TTT}}}{\partial \sigma_{22}}|}_{n}\Delta \sigma_{22}+{\frac{\partial f_{\mathrm{TTT}}}{\partial \sigma_{33}}|}_{n}\Delta \sigma_{33}+2{\frac{\partial f_{\mathrm{TTT}}}{\partial \sigma_{23}}|}_{n}\Delta \sigma_{23}=0$$

Substituting Formula (26) into Formula (28) results in the following:

(29) $$B\Delta \lambda_{\mathrm{IP}}{=f}_{\mathrm{IP}}-C\Delta \lambda_{\mathrm{TTT}}\;F\Delta \lambda_{\mathrm{TTT}}{=f}_{\mathrm{TTT}}-A\Delta \lambda_{\mathrm{IP}}$$

From the above formulas, $\Delta \lambda_{\mathrm{IP}}$ and $\Delta \lambda_{\mathrm{TTT}}$ can be calculated. They can be expressed as

(30) $$\Delta \lambda_{\mathrm{IP}}=\frac{{\mathrm{Ff}}_{\mathrm{IP}}{(\sigma }_{11}^{\mathrm{tri}}{,\sigma }_{12}^{\mathrm{tri}}{,\sigma }_{21}^{\mathrm{tri}}{,\sigma }_{13}^{\mathrm{tri}}{,\sigma }_{31}^{\mathrm{tri}}{)-\mathrm{Cf}}_{\mathrm{TTT}}{(\sigma }_{22}^{\mathrm{tri}}{,\sigma }_{33}^{\mathrm{tri}}{,\sigma }_{23}^{\mathrm{tri}}{,\sigma }_{32}^{\mathrm{tri}})}{\mathrm{BF}-\mathrm{AC}}\;\Delta \lambda_{\mathrm{TTT}}=\frac{{\mathrm{Bf}}_{\mathrm{TTT}}{(\sigma }_{22}^{\mathrm{tri}}{,\sigma }_{33}^{\mathrm{tri}}{,\sigma }_{23}^{\mathrm{tri}}{,\sigma }_{32}^{\mathrm{tri}}{)-\mathrm{Af}}_{\mathrm{IP}}{(\sigma }_{11}^{\mathrm{tri}}{,\sigma }_{12}^{\mathrm{tri}}{,\sigma }_{21}^{\mathrm{tri}}{,\sigma }_{13}^{\mathrm{tri}}{,\sigma }_{31}^{\mathrm{tri}})}{\mathrm{BF}-\mathrm{AC}}$$

where

(31) $$\;{A=D}_{2211}{\frac{\partial f_{\mathrm{TTT}}}{\partial \sigma_{22}}|}_{n}{\frac{\partial f_{\mathrm{IP}}}{\partial \sigma_{11}}|}_{n}{+D}_{3311}{\frac{\partial f_{\mathrm{TTT}}}{\partial \sigma_{22}}|}_{n}{\frac{\partial f_{\mathrm{IP}}}{\partial \sigma_{11}}|}_{n}$$

(32) $$\;{B=D}_{1111}{\left({\frac{\partial f_{\mathrm{IP}}}{\partial \sigma_{11}}|}_{n}\right)}^{2}{+4D}_{1122}{\left({\frac{\partial f_{\mathrm{IP}}}{\partial \sigma_{12}}|}_{n}\right)}^{2}{+4D}_{1313}{\left({\frac{\partial f_{\mathrm{IP}}}{\partial \sigma_{13}}|}_{n}\right)}^{2}$$

(33) $${\;C=D}_{1122}{\frac{\partial f_{\mathrm{IP}}}{\partial \sigma_{11}}|}_{n}{\frac{\partial f_{\mathrm{TTT}}}{\partial \sigma_{22}}|}_{n}{+D}_{1133}{\frac{\partial f_{\mathrm{IP}}}{\partial \sigma_{11}}|}_{n}{\frac{\partial f_{\mathrm{TTT}}}{\partial \sigma_{33}}|}_{n}$$

(34) $${\;F=D}_{2222}{\frac{\partial f_{\mathrm{TTT}}}{\partial \sigma_{22}}|}_{n}{+2D}_{2233}{\frac{\partial f_{\mathrm{TTT}}}{\partial \sigma_{22}}|}_{n}{\frac{\partial f_{\mathrm{TTT}}}{\partial \sigma_{33}}|}_{n}{+D}_{2233}{\left({\frac{\partial f_{\mathrm{TTT}}}{\partial \sigma_{33}}|}_{n}\right)}^{2}$$

Based on the above equations, the stress of CFIT can be updated as follows:

(35) $$\sigma_{11}^{n+1}{=\sigma }_{11}^{\mathrm{tri}}{-D}_{1111}\Delta \lambda_{\mathrm{IP}}{\frac{\partial f_{\mathrm{IP}}}{\partial \sigma_{11}}|}_{n}{-D}_{1122}\Delta \lambda_{\mathrm{TTT}}{\frac{\partial f_{\mathrm{TTT}}}{\partial \sigma_{22}}|}_{n}{+D}_{1133}\Delta \lambda_{\mathrm{TTT}}{\frac{\partial f_{\mathrm{TTT}}}{\partial \sigma_{33}}|}_{n}$$

(36) $$\sigma_{12}^{n+1}{=\sigma }_{21}^{n+1}{=\sigma }_{12}^{\mathrm{tri}}{-2D}_{1212}\Delta \lambda_{\mathrm{IP}}{\frac{\partial f_{\mathrm{IP}}}{\partial \sigma_{12}}|}_{n}$$

(37) $$\sigma_{13}^{n+1}{=\sigma }_{31}^{n+1}{=\sigma }_{13}^{\mathrm{tri}}{-2D}_{1313}\Delta \lambda_{\mathrm{IP}}{\frac{\partial f_{\mathrm{IP}}}{\partial \sigma_{13}}|}_{n}$$

(38) $$\sigma_{22}^{n+1}{=\sigma }_{22}^{\mathrm{tri}}{-D}_{2211}\Delta \lambda_{\mathrm{IP}}{\frac{\partial f_{\mathrm{IP}}}{\partial \sigma_{11}}|}_{n}{-D}_{2222}\Delta \lambda_{\mathrm{TTT}}{\frac{\partial f_{\mathrm{TTT}}}{\partial \sigma_{22}}|}_{n}{-D}_{2233}\Delta \lambda_{\mathrm{TTT}}{\frac{\partial f_{\mathrm{TTT}}}{\partial \sigma_{33}}|}_{n}$$

(39) $$\sigma_{33}^{n+1}{=\sigma }_{33}^{\mathrm{tri}}{-D}_{3311}\Delta \lambda_{\mathrm{IP}}{\frac{\partial f_{\mathrm{IP}}}{\partial \sigma_{11}}|}_{n}{-D}_{3322}\Delta \lambda_{\mathrm{TTT}}{\frac{\partial f_{\mathrm{TTT}}}{\partial \sigma_{22}}|}_{n}{-D}_{3333}\Delta \lambda_{\mathrm{TTT}}{\frac{\partial f_{\mathrm{TTT}}}{\partial \sigma_{33}}|}_{n}$$

(40) $$\sigma_{23}^{n+1}{=\sigma }_{32}^{n+1}{=\sigma }_{23}^{\mathrm{tri}}{-2D}_{2323}\Delta \lambda_{\mathrm{TTT}}{\frac{\partial f_{\mathrm{TTT}}}{\partial \sigma_{23}}|}_{n}$$

## 4. Finite Element Model and Calculation Parameters

### 4.1. Finite Element Model

**Figure 5:**
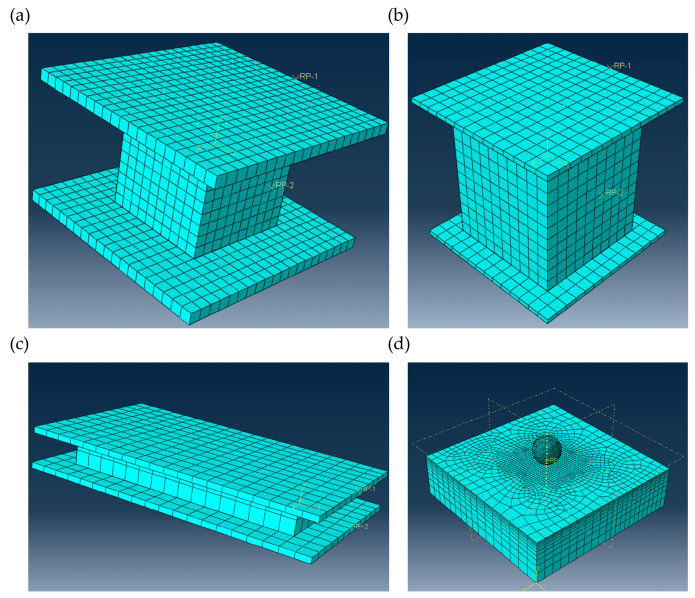
Finite element CFIT models: (**a**) compressive; (**b**) tensile; (**c**) shear; (**d**) indentation.

The above constitutive mode was compiled into a UMAT subroutine using the Fortran language. This UAMT subroutine was then embedded into ABAQUS to calculate the compressive, tensile, shear, and indentation properties of the CFIT and verify the effectiveness of the model. Considering the sample sizes used in the actual tests, finite element models for the compression, tension, shear, and indentation of the material were constructed using ABAQUS, as shown in Figure 5.

Figure 5a–d show the compressive, tensile, shear, and indentation models, respectively, which had corresponding sizes of 20 × 20 × 20 mm^3^, 50 × 50 × 60 mm^3^, 80 × 40 × 10 mm^3^, and 30 × 30 × 8 mm^3^. All loading conditions were consistent with the experiments.

### 4.2. Calculation Parameters

The UMAT subroutine involved 17 property parameters. PROPS(1), PROPS(2), PROPS(8), and PROPS(11) were obtained from the compressive experiments in the IP direction and in the TTT direction. PROPS(5), PROPS(6), PROPS(7), and PROPS(9) were obtained from the shear experiments in different directions. PROPS(10) and PROPS(12) were obtained from the tensile experiments in the IP direction and in the TTT direction. The remaining parameters were derived from empirical values. Their specific values are shown in Table 3.

## 5. Simulation Results and Experimental Verification

### 5.1. Simulation and Verification of Compressive Properties

**Figure 6:**
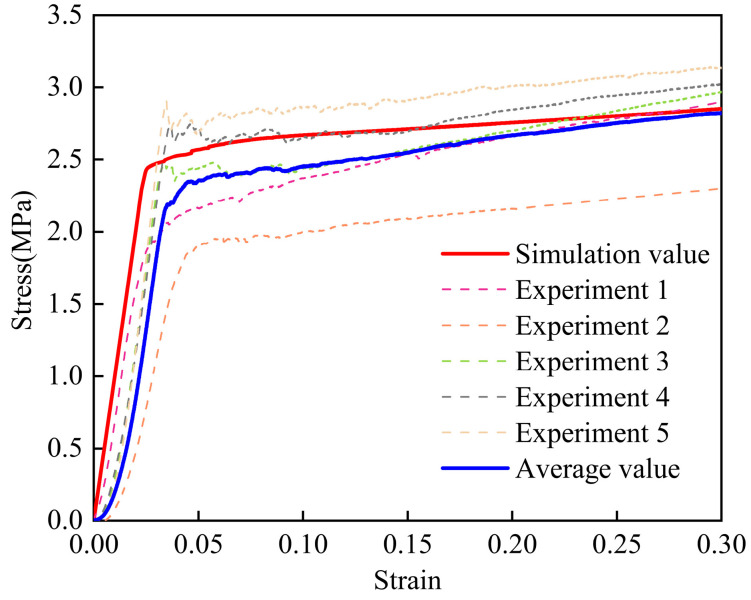
Compressive stress–strain curves in the TTT direction from experiments and calculations.

Figure 6 shows the compressive stress–strain curves in the TTT direction from the calculations and experiments. It can be seen that these had consistent trends for both the calculations and experimental results, showing linear growth in the elastic stage and a platform trend in the plastic stage. The compressive strength of the material obtained by the finite element calculations was 2.49 MPa, having an error of less than 5% when compared with the average experimental value. The calculated elastic modulus of the material was 375 MPa and the error was less than 3% compared with the average experimental value. The final fracture strength of the material was calculated as being about 2.6 MPa and the error in this value was less than 1%.

In summary, compared with the experimental results, the results calculated using the constitutive model had an error of less than 5%. This indicated that the model could accurately reflect changes in the compressive properties of the material, verifying its effectiveness for compressive performance in the TTT direction.

### 5.2. Simulation and Verification of Tensile Properties

**Figure 7:**
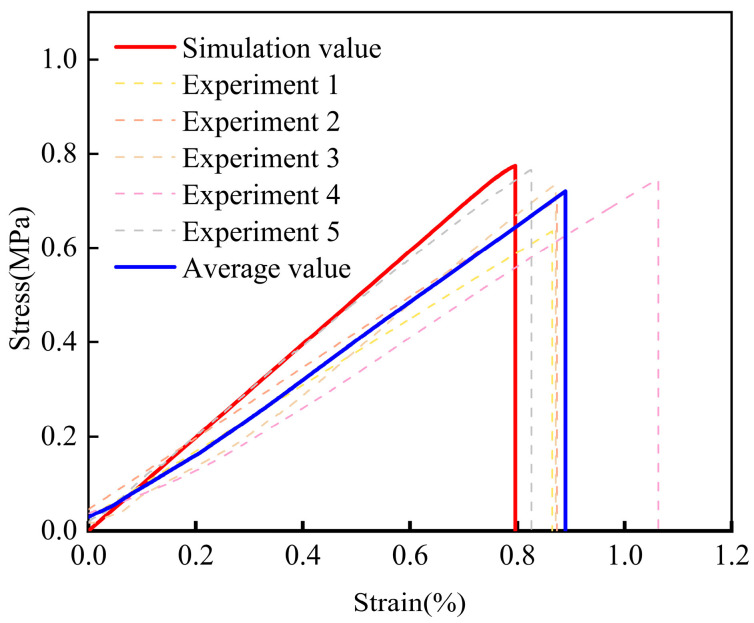
Tensile stress–strain curves in the TTT direction from experiments and calculations.

Figure 7 shows the tensile stress–strain curves in the TTT direction from the calculations and experiments. It can be seen that they all showed brittle fractures that directly occurred after the end of the elastic stage. The calculated tensile strength of the material was 0.75 MPa, which was its breaking strength, and the error in this value was less than 7% when compared with the average experimental value. The tensile elastic modulus of the material was about 95 MPa and the error in this value was less than 18%.

In summary, considering the average experimental values, the error ranges of the values obtained using the constitutive model were from 7% to 18%. This showed that the model could reasonably accurately reflect the changes in the tensile properties of the material in the TTT direction and verified its effectiveness for this purpose.

### 5.3. Simulation and Verification of Shear Properties

**Figure 8:**
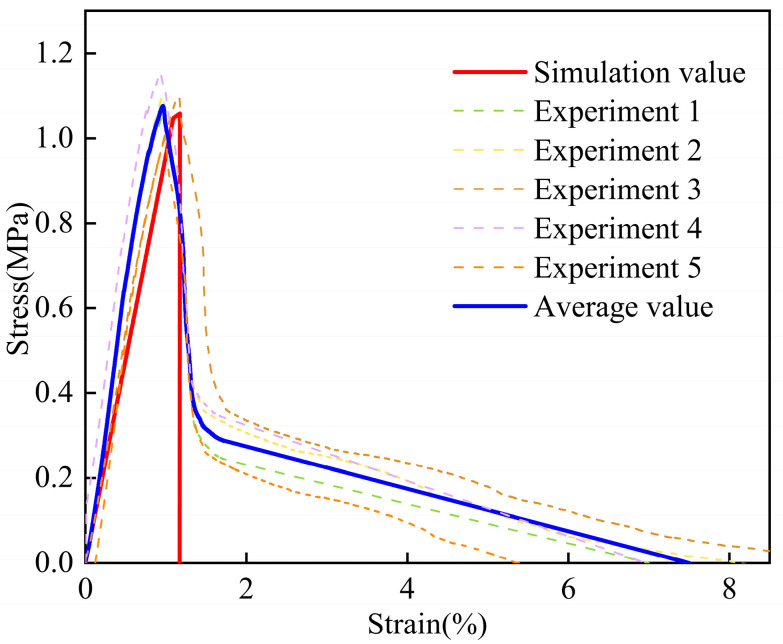
Shear stress–strain curves in the TTT direction from experiments and calculations.

Figure 8 shows the shear stress–strain curves from the calculations and experiments in the TTT direction. From the results of the experiments, brittle fractures occurred in the material and showed a softening trend after it was completely broken. The reason for the phenomenon was that the internal structure of the material had been destroyed, and the friction between the material and the fixture led to a slow decrease in the stress–strain curve. Thus, the results of the shear experiment were equivalent to a brittle fracture, which was the same as the calculated result. The shear strength of the material obtained from the calculations was 1.05 MPa, having an error of less than 1% when compared with the average experimental value. The shear elastic modulus of the material was about 95 MPa and the error in this value was less than 16%.

In summary, considering the experimental results, the error range of the calculation results obtained using the constitutive model was less than 16%. The model could, therefore, accurately reflect changes in the shear properties of the material in the TTT direction, and this verified its effectiveness for this purpose.

### 5.4. Simulation and Verification of Indentation Properties

To verify the reliability and accuracy of the constitutive model, load–displacement curves were produced for the CFITs using indentation experiments under compression and shear loads. The indentation experiments were designed with consideration for the porous characteristics of CFITs. An Instron 5569 universal testing machine was used to determine the mechanical behavior of the material under compression and shear loads at room temperature using a 5 mm Brinell carbide spherical indenter. The size of the samples used for the indentation tests was 30 × 30 × 8 mm^3^.

**Figure 9:**
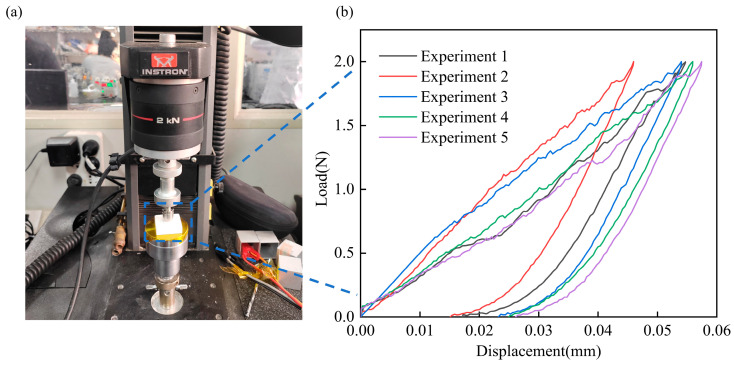
The experimental device and test results of indentation properties: (**a**) experimental device; (**b**) the test result.

The loading and unloading speeds were both 0.001 mm/s and the maximum load was 2 N. The load–displacement curves obtained from these tests are shown in Figure 9. It can be seen that the loading and unloading curves did not overlap, which showed that there was an obvious plastic stage in the CFITs under the effects of pressure and shear. For the TTT direction, the maximum indentation depth in the elastic stage was 0.047 ± 0.007 mm and the minimum indentation depth after unloading was 0.021 ± 0.006 mm.

**Figure 10:**
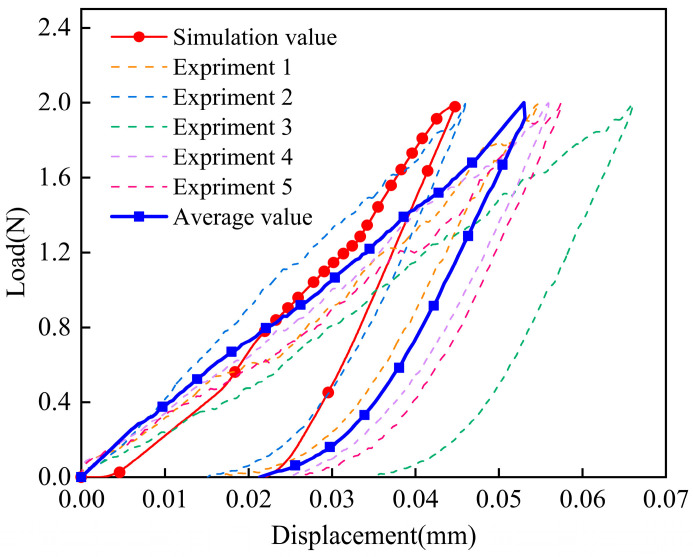
Load–displacement curves of indentation properties in the TTT direction from experiments and calculations.

Figure 10 shows the load–displacement curves of indentation properties in the TTT direction from the experiment and calculations. It can be seen that there was an obvious plastic process in the loading and unloading. The maximum loading depth of the elastic process was 0.045 mm and the error was less than 12%. The minimum unloading depth of the plastic process was 0.022 mm and the error was less than 1%. Considering the experimental results, the error of calculation was within 12%. This showed that the model could effectively reflect the mechanical behavior of the material under compressive and shear loads.

## 6. Conclusions

In this paper, progressive damage factors in the TTT direction were introduced to construct an elastic–plastic damage constitutive model for CFITs by combining the yield criterion and the flow law of transversely isotropic materials. The property parameters involved in the model could be derived from compressive, tensile, and shear tests of the CFIT in different directions. The experimental results of compression, tension, shear, and indentation were consistent with the finite element simulation results, which verified the validity and accuracy of the model. For compression in the TTT direction, the constitutive model was found to have an error of less than 5%. The error ranges of the calculation results for the tensile properties were between 7% and 18% in the TTT direction. For the shear properties, the error range was less than 16%. A ball-indentation experiment was also used to investigate the validity of the model; the maximum loading depth was 0.045 mm and the error of the calculation results was within 12% in the TTT direction. Therefore, this research provides a reliable elastic–plastic damage constitutive model to simulate the mechanical behavior of CFITs under complex working conditions.

## Figures and Tables

**Table 1:** Raw materials and temperature gradient conditions for the preparation of CFITs.

Number	Diameter	Length	Raw Materials	Temperature Gradient
1	1–2 μm	5–6.5 mm	20–30% Alumina fiber	120–170 °C Dry 1200–1300 °C Sintering
2	1–2 μm	5–6.5 mm	40–60% Quartz ceramic fiber	
3	5–8 μm	5–6.5 mm	10–20% Aluminum borosilicate fiber	

**Table 2:** The specific properties of the CFIT.

Performance Index	Value
Density	0.34 g/cm^3^
Compressive strength (in the TTT direction)	2.50 ± 0.51 MPa
Compressive modulus (in the TTT direction)	115.54 ± 16.74 MPa
Compressive strength (in the IP direction)	7.4 ± 0.32 MPa
Compressive modulus (in the IP direction)	380 ± 2.48 MPa
Tensile strength (in the TTT direction)	0.78 ± 0.25 MPa
Tensile modulus (in the TTT direction)	62.46 ± 15.28 MPa
Shear strength (in the TTT direction)	1.12 ± 0.09 MPa
Shear modulus (in the TTT direction)	126.18 ± 15.39 MPa

**Table 3:** Values of input parameters and their physical significance.

Input	Parameter	Value	Physical Meaning
PROPS(1)	E11	380 MPa	Elastic modulus in the IP direction
PROPS(2)	E22	98.8 MPa	Elastic modulus in the TTT direction
PROPS(3)	MU12	0.1	Poisson’s ratio IP in the IP direction
PROPS(4)	MU23	0.1	Poisson’s ratio IP in the TTT direction
PROPS(5)	G12	101 MPa	Shear modulus in the IP direction
PROPS(6)	G23	131 MPa	Shear modulus in the TTT direction
PROPS(7)	XT	1.3 MPa	Shear strength in the IP direction
PROPS(8)	XC	7.6 MPa	Compressive strength in the IP direction
PROPS(9)	XS	1.1 MPa	Shear strength in the TTT direction
PROPS(10)	YT	0.8 MPa	Tensile strength in the TTT direction
PROPS(11)	YC	2.7 MPa	Compressive strength in the TTT direction
PROPS(12)	YS	1.7 MPa	Tensile strength in the IP direction
PROPS(13)	NL	0.1	Coefficient of transfer degree from initial compressive yield surface to final compressive yield surface in the TTT direction
PROPS(14)	NT	0.1	Coefficient of transfer degree from initial compressive yield surface to final compressive yield surface in the IP direction
PROPS(15)	CL	3	Intensity coefficient factor in the TTT direction
PROPS(16)	CT	3	Intensity coefficient factor in the IP direction
PROPS(17)	GHARD	0.1	Hardening factor

## Data Availability

The calculated data from this study can be provided by the corresponding author upon request.
